# Detection and Epidemiological Characterization of Respiratory Pathogens Causing Infections in Patients at Hospital Angeles Lindavista

**DOI:** 10.7759/cureus.97187

**Published:** 2025-11-18

**Authors:** Mauricio Castillo-Salazar, Valentina Flores-Gallardo, María Fernanda Villavicencio-Pérez, José Luis Pinacho-Velázquez, Carlos Andrey Diosdado-Franco, Luis Gerardo Balcázar-Ochoa, Laura Gómez-Virgilio

**Affiliations:** 1 Laboratory and Blood Bank, Hospital Angeles Lindavista, Mexico City, MEX; 2 Medical Education, Hospital Angeles Lindavista, Mexico City, MEX; 3 Pediatrics, Hospital Angeles Lindavista, Mexico City, MEX; 4 General Management, Hospital Angeles Lindavista, Mexico City, MEX

**Keywords:** coinfections, epidemiological characterization, medical comorbidities, respiratory molecular panel, respiratory tract pathogens, risk factors

## Abstract

Background and aim: Respiratory infections are a major cause of morbidity, particularly among young children and older adults. This study aimed to characterize respiratory pathogens detected at Hospital Angeles Lindavista and identify demographic and clinical factors associated with infection patterns.

Methods: This retrospective study analyzed the distribution of respiratory pathogens in patients seen between 2022 and 2024 at a private tertiary hospital using the FilmArray (Lyon, France: bioMérieux) molecular diagnostic panel. We reviewed variables such as demographic data, patient type, comorbidities, risk factors, diagnosis, detected pathogens, and type of infection. We conducted an exploratory analysis using chi-square tests to identify statistically significant associations between our variables (p<0.05). Multinomial regression models were used to characterize these associations.

Results: The dataset included 718 patients, with simple infections being the most common, accounting for 400 (55.6%) cases. The most prevalent pathogens were human rhinovirus/enterovirus (n=117), influenza (n=67), and respiratory syncytial virus (RSV) (n=67). Multivariate regression analysis identified pediatric age as the most significant risk factor for coinfection; children under five years had higher odds of a double infection compared to adults (OR=83.89, 95% CI: 6.38-27531.38, p<0.001). Specific pathogens were strongly age-associated, with RSV and rhinovirus/enterovirus predominating in children, while SARS-CoV-2 was significantly less common in pediatric groups. Notably, a diagnosis of COVID-19 or influenza was negatively associated with the presence of rhinovirus/enterovirus. After model penalization, no statistically significant seasonal pattern was observed.

Conclusion: These findings underscore the importance of age as a major risk factor for respiratory tract infections in general, and for specific etiological agents, such as respiratory syncytial virus, which has particular significance in pediatric populations due to its potential clinical severity and sequelae. These results also highlight the relevance of other microbes, such as human rhinovirus/enterovirus, as a common cause of respiratory tract infections, and previous infections as a risk factor. Finally, this study also emphasizes the potential of molecular diagnostics as a clinical tool and a resource for surveillance, treatment guidance, and preventive strategies.

## Introduction

Respiratory tract infections (RTIs) are a major cause of morbidity and mortality across all age groups [1]. Worldwide, RTIs cause more than 2.5 million deaths [2], and in Mexico, more than 10 million cases are reported annually [3]. While susceptibility to these infections is general, certain groups are more vulnerable than others. Children under the age of five and adults over 65 years are at particularly high risk of becoming infected and developing more severe clinical presentations [1,4]. Therefore, creating a diagnostic process based on each patient's clinical data is vital for accurate diagnosis and effective treatment. However, the similarities in signs and symptoms among different nosological entities, combined with the vast array of potential etiological agents, present clinicians with challenging decisions [1,5].

Until recently, the diagnosis of RTIs relied exclusively on clinical presentation and a limited set of paraclinical tools. Chest X-rays and complete blood counts have long been essential diagnostic aids, though they have significant limitations, non-specificity being among the most prominent. While cultures remain a cornerstone in diagnosing respiratory infections, their major limitations are the narrow spectrum of detectable microorganisms and the time required to obtain results [5,6]. It is also important to note that approximately 70% of RTIs are viral in origin, which restricts the utility of conventional culture techniques [1,4]. Although viral infections are generally self-limiting, under certain risk conditions, such as insufficient breastfeeding in infants or the presence of comorbidities in adults, they can lead to clinical situations that may affect patient safety [4,7]. Moreover, some of these presentations may be caused by atypical bacteria, significantly altering the therapeutic approach required [5,8].

The advent of molecular biology techniques applied to infectious disease diagnostics has enabled the differentiation of these types of infections. This is the case with polymerase chain reaction (PCR)-based panels, which can detect a broad spectrum of viruses and some atypical bacteria. The application of these techniques represents a powerful tool for improving the diagnosis, treatment, and prognosis of patients with RTIs [9,10]. Additionally, the data generated by these tests can help rationalize antibiotic use and identify the seasonal patterns of these infections. This information assists hospitals in developing policies to decrease antibiotic resistance and prevent specific infections on a seasonal basis [9,11-13].

This retrospective study aimed to characterize respiratory pathogens detected in patients at Hospital Angeles Lindavista and identify demographic and clinical factors associated with infection patterns. The results will provide relevant information for developing health strategies and the rational use of antimicrobials within this institution.

## Materials and methods

Study design

This retrospective observational study was conducted at Hospital Angeles Lindavista from January 2022 to July 2024 to characterize the etiological agents in individuals diagnosed with respiratory tract infections. This study included all patients who took a respiratory molecular panel test between January 2022 and July 2024. Patients who did not have the molecular panel report were excluded. Laboratory and clinical data were obtained from the FilmArray (Lyon, France: bioMérieux) multiplex polymerase chain reaction (PCR) system database and electronic medical records through the department of clinical files, with prior authorization from the person in charge of each area, once the protocol had been authorized by the Research Ethics Committee of Hospital Angeles Pedregal. The variables reviewed included demographic data, type of patient (inpatient or outpatient), comorbidities, risk factors, diagnosis, detected pathogens, and type of infection.

The dataset included 718 ambulatory and hospitalized patients, with demographic and clinical variables such as age, sex, patient type, clinical diagnosis, comorbidities, and risk factors. Age groups were classified as children aged under five years, children over five years, adults aged 18-59 years, and older adults aged 60 years and above, according to the classification reported by Matsumura et al. [14]. The information obtained from the variables sought to characterize the study population and its demographic and clinical characteristics.

Laboratory testing

The laboratory analyzed all types of nasopharyngeal samples, including swabs, nasal washes, and aspirates. Respiratory pathogens were detected using the BioFire FilmArray Respiratory Panel v.2.1 (Lyon, France: bioMérieux), a certified multiplex PCR system that integrates sample preparation, amplification, detection, and analysis in a one-hour runtime, enabling rapid results. The panel detects 17 viruses (adenovirus {AdV}; human coronavirus {hCoV} 229E, HKU1, NL63 and OC43; severe acute respiratory syndrome coronavirus 2 {SARS-CoV-2}; influenza A {Inf A} subtypes H1, H1-2009 and H3; influenza B {Inf B}; human metapneumovirus {hMPV}; parainfluenza virus {PIV} type 1, type 2, type 3 and type 4; respiratory syncytial virus {RSV} and rhinovirus/enterovirus {hRV/EV}) and four bacteria (*Bordetella pertussis {B. pertussis}; Bordetella parapertussis {B. parapertussis}; Chlamydophila pneumoniae, and Mycoplasma pneumoniae)*. The test was performed according to the manufacturer's instructions. When the result was valid, it was reported as “detected” or “not detected.” If a failure occurred in the process, the result was labeled “invalid,” which required the test to be repeated. Positive tests were classified based on the number of pathogens detected in the sample as follows: single infections (one pathogen), double infections (two pathogens), and triple infections (three pathogens).

Data source

A retrospective database from the laboratory of Hospital Angeles Lindavista was used. Laboratory and clinical data were obtained from the FilmArray multiplex PCR system database and electronic medical records through the department of clinical files. This database included information from 718 patients collected throughout the study period. It contained clinical and demographic variables. Variable coding was standardized and grouped for statistical analysis.

Statistical analysis

Analyses were performed using R version 4.4.1 (2024-06-14 ucrt) (Vienna, Austria: The R Core Team, R Foundation for Statistical Computing). Frequencies and percentages were used to describe the variables of the study population, and dot plots were used to visualize the number of tests conducted and the proportion of positive results.

An exploratory analysis was conducted using chi-square tests to identify statistically significant associations between the variables. Subsequently, multivariate analyses were conducted using Firth's penalized logistic regression to identify predictors for infection multiplicity, pathogen distribution by age, and pathogen association with clinical diagnosis. For the multivariate regression models, only patients with complete data for all variables included in the model were analyzed. Finally, a t-distributed stochastic neighbor embedding (t-SNE) was applied to reduce the dimensionality of the data and visualize its distribution in a two-dimensional space, thereby exploring potential hidden patterns or clusters among the variables. Statistical significance was defined as p<0.05.

Ethical considerations

The study protocol was approved by the Research Ethics Committee of Hospital Angeles Pedregal (#HAP2787). All procedures were conducted in accordance with the ethical standards outlined in the Declaration of Helsinki. The committee granted a waiver of informed consent for this retrospective study, based on its minimal risk design. Confidentiality and data protection were maintained throughout the study.

## Results

Clinical and demographic characteristics

The findings showed the population exhibited a slight majority of males (405, 56.4%) over females (313, 43.6%). The most representative age group was children aged <5 years (323, 45%), with a median age of two years. Clinically, most individuals required hospitalization (591, 82.3%). Regarding clinical diagnosis, pneumonia was the most common (211, 29.4%), whereas 90.5% (651) of patients recorded no comorbidities. The most frequent comorbidities were insulin resistance or type 2 diabetes mellitus (26, 3.6%) and arterial hypertension (21, 2.9%). As for risk factors, the most common category was “not indicated” (488, 67.9%), followed by “other” (71, 9.9%), short or absent breastfeeding (55, 7.6%), and prematurity (37, 5.1%). Full characteristics are presented in Table 1.

**Table 1:** Characteristics of the study population. The analysis of the studied variables identified a male majority and children <5 years as the most represented age group. While most individuals required hospitalization, pneumonia was identified as the most common clinical diagnosis, and insulin resistance or type 2 diabetes mellitus and short or absent breastfeeding were reported as the most frequent comorbidities and risk factors, respectively.

Variables	Category	Frequency	%
Gender	Male	405	56.41
	Female	313	43.59
Age group (n, median)	Children <5 years	323 (2)	44.99
	Children >5 years	158 (8)	22.00
	Adults 18-59 years	164 (37)	22.84
	Adults ≥60 years	73 (75)	10.17
Patient type	Outpatient	127	17.69
	Hospitalized	591	82.31
Clinical diagnosis	Pneumonia	211	29.39
	Others	189	26.32
	Upper respiratory tract infection	79	11.00
	No diagnosis	61	8.49
	Bronchitis	47	6.55
	Influenza	46	6.41
	Laryngotracheitis	16	2.23
	Rhinopharyngitis	16	2.23
	Bronchiolitis	15	2.09
	COVID-19	13	1.81
	Gastroenteritis	13	1.81
	Asthma	12	1.67
Comorbidities	Not indicated	651	90.67
	Insulin resistance/type 2 diabetes mellitus	26	3.62
	Arterial hypertension	21	2.92
	Pulmonary disease	14	1.95
	Other	6	0.84
Risk factors	Not indicated	488	67.97
	Other	71	9.89
	Short or absent breastfeeding	55	7.67
	Prematurity	37	5.15
	Systemic arterial hypertension	22	3.06
	Previous viral infections	12	1.67
	Allergies	10	1.39
	Chronic kidney disease (CKD)	8	1.11
	Hypothyroidism	8	1.11
	Lack of vaccination	7	0.97

Distribution of infections

The analysis of the population by age group showed that simple infections and coinfections were more frequent in children aged under five years than in any other age group (Table 2). In this group, 32.7% (235) of individuals were infected by a single pathogen. After children under five years of age, children over five years followed in frequency of infections, with 84 (11.7%) individuals. Adults aged 18-59 years and adults aged over 60 years followed with 59 (8.2%) and 22 (3.1%) subjects, respectively. Coinfections (double and triple) were also more prevalent among children under five years of age, suggesting susceptibility to multiple infections in this population.

**Table 2:** Distribution of infections type by age group. The table shows the number of single, double and triple infections across different age categories. The total per group represents the sum of all infection types within each age group, while the total per infection type represents the sum across all age categories.

Age groups	Single infection	Dual infection	Triple infection	Total per group
Children <5 years	235	36	3	274
Children >5 years	84	17	0	101
Adults 18-59 years	59	4	1	64
Adults ≥60 years	22	0	0	22
Total per infection type	400	57	4	461

Examination of the number of pathogens detected by infection, regardless of age group, showed that, overall, 400 (55.6%) patients had a single infection, and 257 (35.8%) showed no microbiological isolation, due to low viral load, non-viral infections, or late sample collection. Coinfections accounted for only 8.5% (60) of all cases, whereas double infections accounted for 56 (7.9%) cases, and triple infections accounted for four (0.6%) cases (Table 2).

Etiologic agents

BioFire FilmArray respiratory panel detects 17 viruses and four bacteria. The following section describes the detected viruses in both single and double infections, as well as in cases where more than three microorganisms were identified by the molecular panel.

Among single-infection cases (n=400), the most frequent agents were rhinovirus/human enterovirus (117, 29.25%), influenza (67, 16.75%), respiratory syncytial virus (RSV) (57, 14.25%), human metapneumovirus (49, 12.25%), and parainfluenza virus (38, 9.5%). Other agents such as SARS-CoV-2, adenovirus, coronavirus, and *Mycoplasma pneumoniae* were less prevalent (Table 3). In cases of double infections (n=56), the most frequently observed combinations included RSV and human rhinovirus/enterovirus (9, 16%), parainfluenza virus and human rhinovirus/enterovirus (7, 12.5%), and RSV and adenovirus (6 cases). Complex pathogen profiles were identified in fewer infections (n=4), such as the triple combination of respiratory syncytial virus+parainfluenza virus+human rhinovirus/enterovirus.

**Table 3:** Frequency of detected pathogens according to type of infection. The table summarizes the microorganisms identified in single, double, and triple infections detected by the molecular respiratory panel.

Variables	Pathogen or combination	Frequency (n)
No infection	-	257
Single infection	Human rhinovirus/enterovirus	117
	Influenza	67
	Respiratory syncytial virus	57
	Human metapneumovirus	49
	Parainfluenza	38
	SARS-CoV-2	29
	Adenovirus	27
	Seasonal coronavirus	15
	Mycoplasma pneumoniae	1
	Total	400
Dual infection	Respiratory syncytial virus+human rhinovirus/enterovirus	9
	Parainfluenza+human rhinovirus/enterovirus	7
	Respiratory syncytial virus+adenovirus	6
	Human rhinovirus/enterovirus+adenovirus	6
	Influenza+others	4
	Human rhinovirus/enterovirus+others	4
	Human rhinovirus/enterovirus+coronavirus	4
	Parainfluenza+others	3
	Influenza+coronavirus	3
	SARS-CoV-2+coronavirus	3
	Respiratory syncytial virus+others	3
	Human metapneumovirus+coronavirus	2
	*Bordetella pertussis*+human rhinovirus/enterovirus	1
	*Bordetella parapertussis*+respiratory syncytial virus	1
	*Bordetella parapertussis*+human rhinovirus/enterovirus	1
	Total	57
Triple infection	Respiratory syncytial virus+parainfluenza+human rhinovirus/enterovirus	2
	Parainfluenza+human rhinovirus/enterovirus+adenovirus	1
	SARS-CoV-2+coronavirus+adenovirus	1
	Total	4

Seasonality

**Figure 1:**
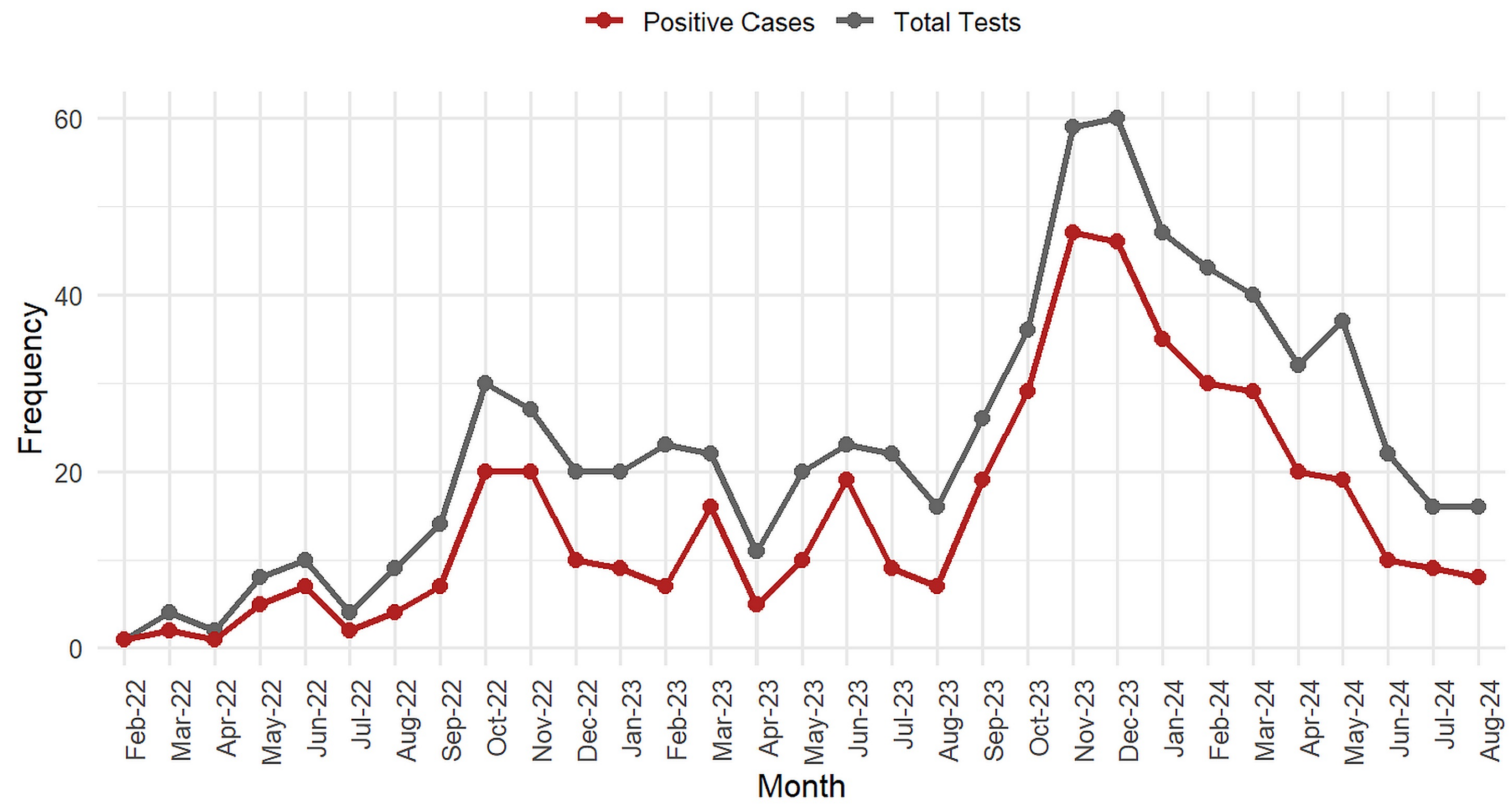
Seasonal trend in respiratory virus testing and positivity (February 2022-August 2024). There were two prominent seasonal waves of positive cases during autumn and winter. The first wave peaked in late 2022, while the second one occurred in late 2023, suggesting periods of increased viral circulation.

Another variable analyzed was the date of the test. The results were grouped monthly, grouped by positive cases and total tests. The monthly evolution of tests performed and positive cases showed a progressive increase during the second half of each year. The highest number of tests was recorded in November 2023 (n=60), with 47 positive cases (78.3%). This trend declined in the early months of 2024 (Figure 1).

**Figure 2:**
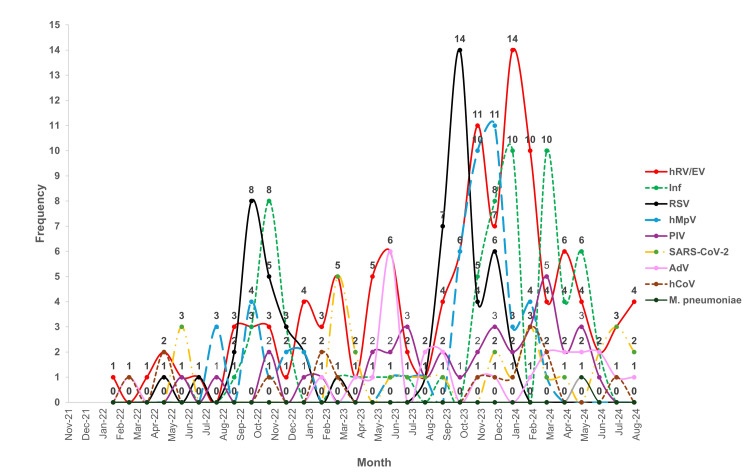
Seasonal trends of respiratory pathogens detected in simple infections. Frequencies of positive sampling of specific pathogens by month. hRV/EV: human rhinovirus/enterovirus; Inf: influenza; RSV: respiratory syncytial virus; hMPV: human metapneumovirus; PIV: parainfluenza virus; SARS-CoV-2: severe acute respiratory syndrome coronavirus 2; ADV: adenovirus, HCOV: human coronavirus, *M. pneumoniae*: *Mycoplasma pneumoniae*

Regarding the distribution of pathogens detected in simple infections, human rhinovirus/enterovirus (hRV/EV) was the most frequently identified throughout the analyzed period, peaking in January 2024 (red line, Figure 2). Influenza followed in prevalence, with four peaks observed as follows: November 2022, January, March, and May 2024 (green dotted line, Figure 2). RSV, the third most detected pathogen in simple infections, showed two peaks, suggesting seasonal variation (black line, Figure 2). hMPV exhibited a notable peak in December 2023 (blue dotted line, Figure 2), while parainfluenza virus (PIV) showed consistent positivity from May 2023 onward, with a slight increase in March 2024 (purple line, Figure 2). SARS-CoV-2, the most frequently reported pathogen in simple infections among subjects over 60 years old, peaked in March 2023 (gold dotted line, Figure 2). Adenovirus (AdV) peaked in June 2023 and remained consistently positive from March to June 2024, indicating seasonality (pink line, Figure 2). Lastly, positive cases of hCoV mostly occurred in February 2023 and 2024 (brown dotted line, Figure 2).

Exploratory analysis

Once we described demographic characteristics and etiological agents, we sought to determine if there was an association between the type of infection and clinical variables. First, we performed an exploratory analysis through chi-square testing. Chi-square tests identified significant associations between the type of infection and variables, such as age group, clinical diagnosis, comorbidities, and risk factors (p<0.05). No associations were observed with gender or patient type (Table 4).

**Table 4:** Association between clinical variables and type of infection. Results from the chi-square test show p-values for each variable. A significant p-value (<0.05) was identified for the first four variables, whereas no significant association was found for the last two.

Variables	Chi-square	p-Value
Age group	151.37	2.99×10^-26 ^→ <0.05
Clinical diagnosis	209.84	2.07×10^-17 ^→ <0.05
Comorbidities	52.89	7.85×10^-6 ^→ <0.05
Risk factors	57.1	1.41×10^-2 ^→ <0.05
Gender	4.65	3.25×10^-1 ^→ >0.05
Patient type	4.28	3.69×10^-1 ^→ >0.05

Multivariate analysis

Considering variables that were significant in the exploratory analysis, multivariate analyses were conducted using Firth's penalized logistic regression to identify predictors for infection multiplicity, pathogen distribution by age, and pathogen association with clinical diagnosis.

Factors associated with infection multiplicity

**Figure 3:**
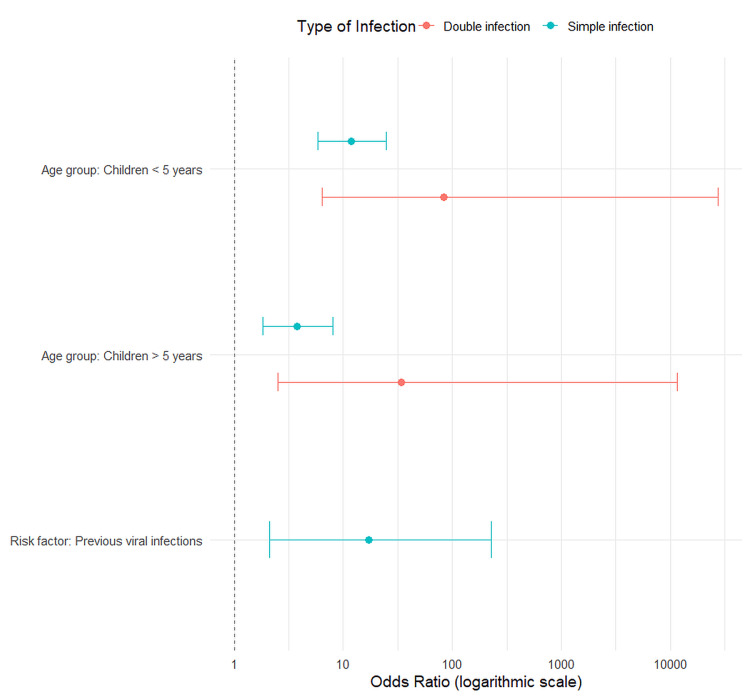
Factors associated with simple and double respiratory infections. The forest plot displays odds ratios (points) and 95% confidence intervals (horizontal lines) from Firth's penalized logistic regression model. The analysis identified factors associated with an increased risk of a simple or double infection compared to no infection. The vertical dashed line indicates no effect. Only statistically significant associations (p<0.05) are shown.

First, we identified factors associated with simple versus double infections (Figure 3). Pediatric age was the most significant predictor when compared to the adult reference group (18-59 years). Children under five years showed the highest odds for both simple (OR=11.86, 95% CI: 5.89-24.90, p<0.001) and double infections (OR=83.89, 95% CI: 6.38-27531.38, p<0.001). Children over five years also had significantly increased odds for simple (p<0.001) and double infections (p=0.003). Interestingly, previous viral infections increased the likelihood of a simple infection (OR=17.16, 95% CI: 2.09-229.20, p=0.007), but not a double one (p=0.261). We found no other comorbidities or risk factors with a statistically significant difference.

Association of pathogens with patient age

**Figure 4:**
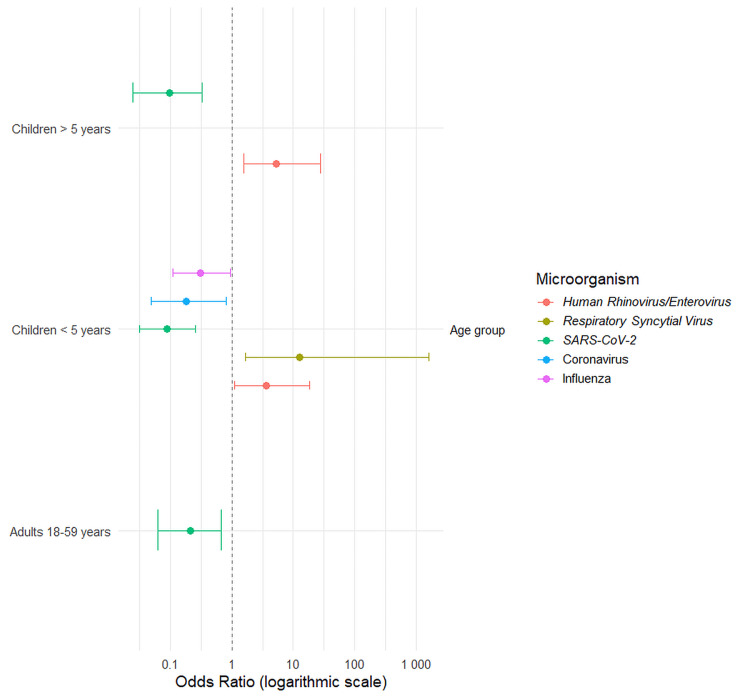
Association between age group and etiological agents in simple respiratory infections. The forest plot shows odds ratios (OR) with 95% confidence intervals (CI) from a logistic regression model, using adults (18-59 years) as the reference group. The vertical dashed line at an odds ratio of 1.0 indicates no effect. Only statistically significant associations (p<0.05) are shown.

We assessed the association between specific pathogens and patient age, using individuals over 59 years as the reference group (Figure 4). Respiratory syncytial virus (RSV) was strongly associated with children under five years of age (OR=12.51, 95% CI: 1.68-1598.8, p=0.007), while* *human rhinovirus/enterovirus was associated with both groups of children (p=0.032; p=0.006). SARS-CoV-2 showed a negative association with all younger age groups, but a higher odds in the >59 years reference group. Infections with seasonal coronavirus (p=0.026) and* *influenza(p=0.041) were less frequent in children under five years of age. Adenovirus, human metapneumovirus, *Mycoplasma pneumoniae*, and parainfluenza virus showed no significant age-based associations.

Association of pathogens with clinical diagnosis

**Figure 5:**
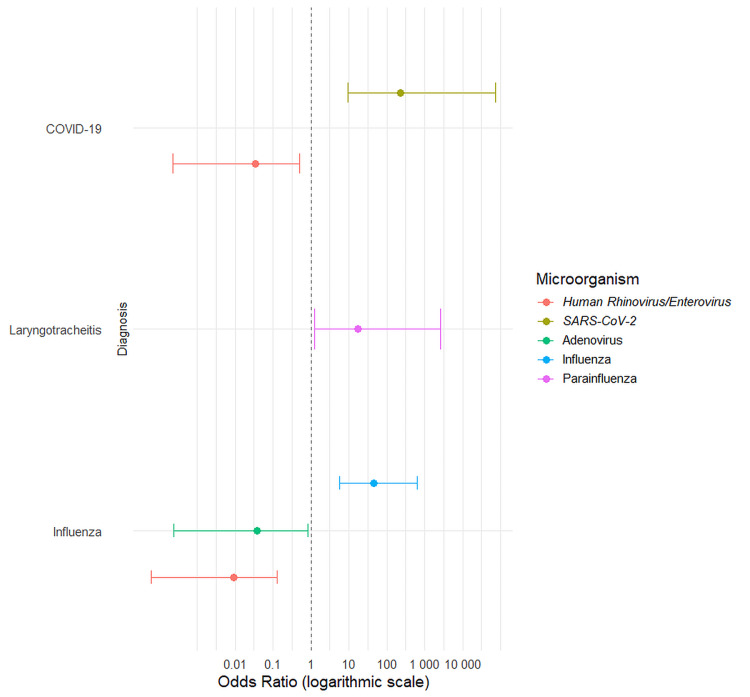
Association between respiratory pathogens and specific clinical diagnoses. This forest plot shows the odds ratios (OR) with 95% confidence intervals (CI) from a logistic regression analysis, assessing the odds of detecting a pathogen in patients with a specific clinical diagnosis versus those without. The vertical dashed line at an odds ratio of 1.0 indicates no effect. Only statistically significant associations (p<0.05) are shown.

We then evaluated associations between pathogens and specific clinical diagnoses (Figure 5). There were some expected associations, for instance, SARS-CoV-2 with a COVID-19 diagnosis (OR=231.0, p<0.001), influenza virus with influenza diagnosis (OR=45.0, p<0.001), and parainfluenza virus with laryngotracheitis (OR=17.29, p=0.030). The analysis also revealed significant negative associations. The detection ofhuman rhinovirus/enterovirus was linked to substantially lower odds of a concurrent diagnosis of either COVID-19 (p=0.012) or influenza (p<0.001). A similar negative association was found between adenovirus and an influenza diagnosis (p=0.037).

Seasonal influence on pathogen distribution

Finally, a Firth's penalized logistic regression model was employed to determine if the detection of specific pathogens was associated with a particular season, using Spring as the reference period. The analysis did not reveal any statistically significant association between any of the studied etiological agents and the seasons of the year.

Dimensionality reduction

A t-distributed stochastic neighbor embedding (t-SNE) analysis was applied to identify clustering of data (data not shown). However, no clear separation was observed between infection types, suggesting a high degree of overlap between the groups.

## Discussion

This retrospective study characterized the distribution of respiratory pathogens in pediatric and adult patients treated at a private tertiary care hospital between 2022 and 2024, using the FilmArray molecular diagnostic platform. Our findings confirm that single respiratory infections are the most frequent across all age groups, particularly in children under five years of age, who also exhibited higher rates of coinfections (both dual and triple).

The high prevalence of infections caused byhuman rhinovirus/enterovirus, influenza, and respiratory syncytial virus(RSV) is consistent with prior literature and reinforces their role as the primary etiological agents in respiratory illnesses [15]. Furthermore, our results show frequent combinations of RSV with other viruses in dual infections, which have important clinical implications, as coinfections may be associated with greater severity or prolonged symptoms [16,17].

Our analysis with Firth's penalized regression identified pediatric age as the most critical factor associated with both simple and double respiratory infections. The odds of a double infection were significantly higher in children under five years of age (OR≈84) and those over five years (OR≈34) compared to adults. This finding is congruent with established knowledge regarding immunological immaturity and increased pathogen exposure common in childhood [18-20]. This susceptibility is further detailed by our pathogen-specific findings, which demonstrate that the risk is higher for viruses like RSV and human rhinovirus/enterovirus (Figure 4) [21].

Interestingly, a history of previous viral infections was significantly associated with simple infections, suggesting that recent immune challenges may alter susceptibility, though this effect was not observed for double infections [22]. Notably, after Firth's penalization, many factors traditionally regarded as risk factors, such as vaccination status, comorbidities, prematurity, or breastfeeding history, did not show a statistically significant association in our model (data not shown). While this may suggest that their individual impact may be smaller than age, these results should be interpreted cautiously due to the predominance of children in the cohort.

Surprisingly, our study did not find a statistically significant seasonal association for any specific pathogen. Even though respiratory viruses can surge in autumn and winter, the lack of a clear pattern in our data could be attributed to several factors, including regional climatic conditions or the conservative nature of the penalized regression model, which is designed to avoid overstating potentially spurious associations [20,23].

Our analysis of pathogen diagnosis associations confirmed expected links (e.g., SARS-CoV-2 with COVID-19 and influenza virus with influenza) but also revealed significant negative associations (Figure 5). For instance, human rhinovirus/enteroviruswas less likely to be present during a diagnosed case of COVID-19 or influenza. One interpretation of these findings is that clinical judgement is a good first lead in diagnosis and therapeutic decision-making. Another possible explanation for negative associations is viral interference, a phenomenon where infection with one virus inhibits coinfection by another, which is plausible [24]. Finally, dimensionality reduction through t-SNE was applied to explore the structure of clinical variables. Nonetheless, no clustering was observed, which may stem from the heterogeneity of the variables.

Overall, these findings highlight the value of multiplex molecular testing not only as a diagnostic tool but also as a rich source of epidemiological data for surveillance and clinical decision-making. They also emphasize the importance of contextualizing results according to demographic, clinical, and seasonal variables. In the future, integrating these data with detailed clinical records and follow-up outcomes could improve prognosis and support personalized care for patients with respiratory infections.

This study has several limitations that should be considered. First, as a retrospective study based on existing laboratory records, we had limited control over data completeness. Some relevant clinical variables, such as symptom duration or detailed vaccination history, were frequently unavailable, with missing data occurring in approximately 20% of cases for these items. We handled missing data through a complete-case analysis for the multivariate models. This approach carries the risk of selection bias if the missingness is not random. For example, if patients with unavailable vaccination records were systematically different from those with complete records (e.g., differing in socioeconomic status), our estimates of vaccine effectiveness could be biased. Therefore, while our results are robust for the population with complete data, they should be extrapolated to the general population carefully. We underscore the need for prospective studies designed to ensure the complete collection of this critical information.

Furthermore, as this is a single-center study conducted in a private tertiary hospital in Mexico City, the findings may not be generalizable to public healthcare systems or other geographic regions. Additionally, although we attempted to mitigate the effects of variable overfitting through penalization in the statistical analysis, this approach may have overlooked data trends with potentially clinically relevant information.

Finally, this study is constrained by the diagnostic panel used (FilmArray) and its qualitative nature. The panel does not measure viral load, making it impossible to distinguish between active infection and asymptomatic carriage. Lastly, the study period (2022-2024) reflects a unique post-pandemic era of viral circulation, which may differ from historical epidemiological patterns.

## Conclusions

These findings underscore age as a major risk factor for respiratory tract infections in general, and for specific etiological agents, such as respiratory syncytial virus*,* which has a particular importance in pediatric populations due to potential clinical severity and sequelae. These results also highlight the relevance of other microbes, like human rhinovirus/enterovirus*, *as a very common cause of respiratory tract infections, and previous infections as a risk factor for new infections. Finally, this study also emphasizes the potential of molecular diagnostics as a clinical tool and a resource for surveillance, treatment guidance, and preventive strategies.

## References

[1] García JL, Hertera AM (2015). Infección de vías respiratorias agudas en población pediátrica. [Article in Spanish]. Rev Enferm Infecc Pediatr.

[2] Ni X, Kang N, Huang K (2025). The global burden of pediatric respiratory infections attributable to long-term exposure to fine particulate matters. Environ Pollut.

[3] (2025). Informe semanal infección respiratoria aguda (IRA). https://www.gob.mx/cms/uploads/attachment/file/1025041/Informesemanal_IRA_SE37.pdf.

[4] Chu FL, Li C, Chen L, Dong B, Qiu Y, Liu Y (2022). Respiratory viruses among pediatric inpatients with acute lower respiratory tract infections in Jinan, China, 2016-2019. J Med Virol.

[5] Gentilotti E, De Nardo P, Cremonini E (2022). Diagnostic accuracy of point-of-care tests in acute community-acquired lower respiratory tract infections. A systematic review and meta-analysis. Clin Microbiol Infect.

[6] Calderaro A, Buttrini M, Farina B, Montecchini S, De Conto F, Chezzi C (2022). Respiratory tract infections and laboratory diagnostic methods: a review with a focus on syndromic panel-based assays. Microorganisms.

[7] Alverca-Ordóñez N, Samaniego-Luna N, Jaramillo VM (2022). Lactancia materna como factor protector de infecciones respiratorias altas. CEDAMAZ.

[8] Barros PB, Xavier LF, Herter ED, Fernandes MF, Ferreira IC, Pinto LA (2024). Atypical bacterial respiratory infections in children. J Bras Pneumol.

[9] Hernández-González DG, Rodríguez-Muñoz L, Solórzano-Santos F (2021). Impact of the use of multiplex PCR on etiological diagnosis and treatment of acute respiratory infections in a private hospital of the north of the country. Gac Med Mex.

[10] Zheng Y, Qiu X, Wang T, Zhang J (2021). The diagnostic value of metagenomic next-generation sequencing in lower respiratory tract infection. Front Cell Infect Microbiol.

[11] Barry B, Bernard S (2018). Infecciones de las vías respiratorias superiores. EMC-Tratado de Medicina.

[12] Xie LY, Wang T, Yu T (2024). Seasonality of respiratory syncytial virus infection in children hospitalized with acute lower respiratory tract infections in Hunan, China, 2013-2022. Virol J.

[13] Maison N, Omony J, Rinderknecht S (2024). Old foes following news ways? - Pandemic-related changes in the epidemiology of viral respiratory tract infections. Infection.

[14] Matsumura Y, Yamamoto M, Tsuda Y (2025). Epidemiology of respiratory viruses according to age group, 2023-24 winter season, Kyoto, Japan. Sci Rep.

[15] Khales P, Razizadeh MH, Ghorbani S, Moattari A, Saadati H, Tavakoli A (2025). Prevalence of respiratory viruses in children with respiratory tract infections during the COVID-19 pandemic era: a systematic review and meta-analysis. BMC Pulm Med.

[16] Hayek H, Amarin JZ, Qwaider YZ (2023). Co-detection of respiratory syncytial virus with other respiratory viruses across all age groups before and during the COVID-19 pandemic. Front Virol.

[17] Georgakopoulou VE (2024). Insights from respiratory virus co-infections. World J Virol.

[18] Mandelia Y, Procop GW, Richter SS, Worley S, Liu W, Esper F (2021). Dynamics and predisposition of respiratory viral co-infections in children and adults. Clin Microbiol Infect.

[19] Nieto-Rivera B, Saldaña-Ahuactzi Z, Parra-Ortega I (2023). Frequency of respiratory virus-associated infection among children and adolescents from a tertiary-care hospital in Mexico City. Sci Rep.

[20] Simon AK, Hollander GA, McMichael A (2015). Evolution of the immune system in humans from infancy to old age. Proc Biol Sci.

[21] Leija-Martínez JJ, Cadena-Mota S, González-Ortiz AM (2024). Respiratory syncytial virus and other respiratory viruses in hospitalized infants during the 2023-2024 winter season in Mexico. Viruses.

[22] Rakebrandt N, Joller N (2019). Infection history determines susceptibility to unrelated diseases. Bioessays.

[23] Li X, Ma J, Li Y, Hu Z (2025). One-year epidemiological patterns of respiratory pathogens across age, gender, and seasons in Chengdu during the post-COVID era. Sci Rep.

[24] Cai M, Xu E, Xie Y, Al-Aly Z (2025). Rates of infection with other pathogens after a positive COVID-19 test versus a negative test in US veterans (November, 2021, to December, 2023): a retrospective cohort study. Lancet Infect Dis.

